# Circulating tumor DNA predicts tumor recurrence in early-stage breast cancer: A meta-analysis

**DOI:** 10.1016/j.gendis.2025.101908

**Published:** 2025-10-30

**Authors:** Luke F. Moat, John G. Mayer, David S. Puthoff, Adam M. Bissonnette, Abdul R. Shour, Scott J. Hebbring, Adedayo A. Onitilo, Zhi Wen

**Affiliations:** a Center for Precision Medicine Research, Sanford-Marshfield Clinic Research Institute, Marshfield Clinic Health System, Marshfield, WI 54449, USA; b Office of Research Computing and Analytics, Sanford-Marshfield Clinic Research Institute, Marshfield, WI 54449, USA; c Cancer Care and Research Center, Sanford-Marshfield Clinic Research Institute, Marshfield Clinic Health System, Marshfield, WI 54449, USA; d Integrated Research & Development Lab, Sanford-Marshfield Clinic Research Institute, Marshfield Clinic Health System, Marshfield, WI 54449, USA

The annualized rate of breast cancer recurrence is 10.4% within the first five years after surgery, with the highest risk (15.2%) occurring during the first two years.^1^ Despite the integration of mammography, MRI, CT, and PET scans into follow-up protocols, recurrence remains the leading cause of breast cancer–related mortality, accounting for an estimated 42,250 deaths in the United States in 2024. Early detection of tumor recurrence is therefore critical to improving the prognosis of breast cancer patients by enabling timely and targeted therapies. Circulating tumor DNA (ctDNA), which originates from tumor cells and enters the bloodstream (Fig. S1), has emerged as a promising biomarker for real-time monitoring of tumor burden. ctDNA is typically double-stranded and shorter than 200 nucleotides. Notably, its half-life ranges from 16 min to 2.5 h, supporting its utility as a dynamic biomarker.^2^ Measuring ctDNA in blood samples has evolved into a non-invasive liquid biopsy approach capable of predicting tumor recurrence prior to clinical diagnosis. Here, we performed a meta-analysis to evaluate ctDNA as a biomarker for early-stage breast cancer recurrence, with the goal of supporting its integration into clinical decision-making.

Firstly, we summarized the ctDNA detection techniques applied in early-stage breast cancer studies in our meta-analysis, as outlined in the PRISMA 2020 Checklist (Fig. 1A; Fig. S2 and Supplemental Methods). ctDNA assays were categorized as either informed or naïve and employed PCR-based or next-generation sequencing (NGS) approaches (Fig. S3). Informed assays help eliminate background noise caused by clonal hematopoiesis of indeterminate potential, whereas naïve assays offer shorter turnaround times. We identified 16 informed and 5 naïve studies detecting ctDNA in plasma samples collected during the follow-up period (Table S1). Among the informed studies, 6 used ddPCR to detect ctDNA at multiple time points, including baseline at diagnosis (T0), post-neoadjuvant treatment (T1), post-surgery (T2), and during adjuvant therapy or follow-up (T3). These studies typically tracked 1–2 variants per patient, though up to 19 variants were monitored in some cases. The informed group also included assays such as Signatera (*n* = 4), RaDaR (*n* = 2), NeXT Personal Dx (*n* = 1), Invitae (*n* = 1), and two in-house NGS platforms, all of which tracked multiple variants simultaneously. In contrast, the naïve group included Guardant Reveal (*n* = 2), FoundationOne Liquid (*n* = 1), ddPCR (*n* = 1), custom NGS assay (*n* = 1), and tTDS assay (*n* = 1).

**Figure 1:**
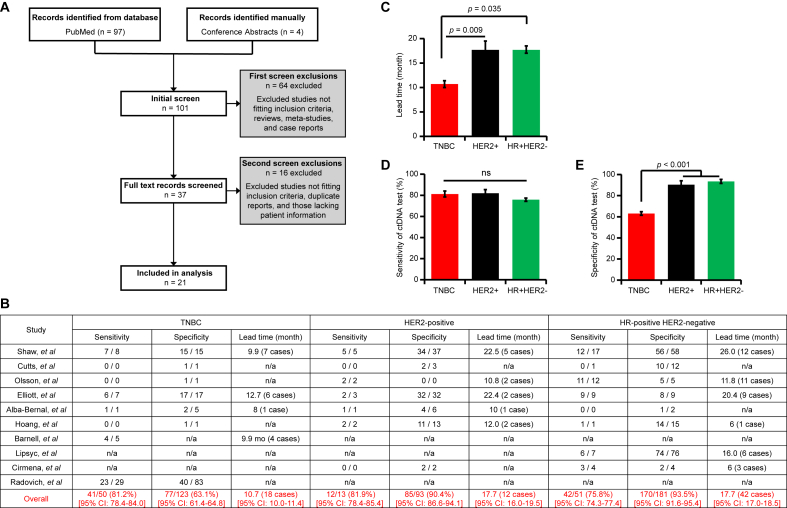
The meta-analysis of ctDNA testing results in the follow-up periods of post-surgery early-stage breast cancer patients demonstrates better performance of ctDNA testing in the HER2^+^ and HR^+^HER2^−^ subtypes compared to the TNBC subtype. **(A)** The PRISMA flow chart outlines the literature search, selection, and exclusion process. **(B)** The average sensitivity, specificity, and lead time of ctDNA assays for each cohort are shown. Averages with 95% Confidence Intervals (CIs) were calculated using Python's NumPy, weighted by sample size or case number in each study. **(C)** A two-tailed meta-analysis was conducted to compare lead times between the TNBC, HER2^+^, and HR^+^HER2^−^ cohorts, accounting for unequal variances. **(D–E)** A two-tailed chi-square test with two degrees of freedom was performed to compare the sensitivity (C) and specificity (D) of ctDNA testing between the three cohorts. Mean values with 95% CIs are presented in (C–E).

Secondly, ctDNA levels are dynamic and closely correlate with changes in tumor burden. Detection rates at T0, T1, and T2 varied in accordance with the clinical status of the primary tumor during curative treatment. In contrast, ctDNA detection during the follow-up period is more appropriate for predicting tumor recurrence, as the primary tumor has already been surgically removed. All 21 included studies collected blood samples during the follow-up period, in addition to other time points. Therefore, we recalculated the sensitivity and specificity for each ctDNA study based solely on the raw data from the follow-up period (Table S1).

Thirdly, the number of blood draws during the follow-up period varied across studies, ranging from a single draw to multiple draws. Detecting ctDNA at multiple time points introduced a statistical challenge in calculating lead time. We defined lead time as the interval between the first ctDNA detection and the clinical confirmation of tumor recurrence and used it to evaluate the predictive potential of ctDNA. Notably, the frequency of blood draws during follow-up varied—occurring every 2, 3, 6, or 12 months (Table S1)—which could influence the observed lead time. Among 14 studies that reported this metric, the recalculated lead time ranged from 3.3 to 20.3 months, potentially providing clinicians with a window of opportunity to intervene and manage residual disease to prevent tumor recurrence.

Fourthly, triple-negative breast cancer (TNBC) is associated with a higher risk of distant recurrence than other molecular subtypes (HR 2.6; 95% CI: 2.0–3.5; *p* < 0.0001).^3^ Magbanua, et al, reported a higher rate of ctDNA positivity in TNBC compared to HR^+^HER2^−^ using the Signatera assay.^4^ Among the 21 studies, 20 described the tumor's molecular subtypes: 4 studies of TNBC only, 2 studies of HR^+^HER2^-^ type only, and 14 studies of mixed molecular subtypes (Table S1).

Next, we selected 12 studies carrying follow-up data to evaluate the overall performance of ctDNA testing, excluding those conducted in highly-overlapped patient cohorts or with other ineligibilities (Table S2). Across these studies, we observed an average sensitivity of 80.7% (116/146 cases; 95% CI: 80.3%–81.1%), an average specificity of 78.1% (428/504 cases; 95% CI: 77.6%–78.5%), and an average lead time of 15.5 months (77 cases; 95% CI: 15.1–15.9) for ctDNA in predicting tumor recurrence in post-surgical early-stage breast cancer. Despite our efforts, we were unable to summarize the performance of specific ctDNA platforms across studies, as they were conducted in different patient cohorts. However, two studies by Drs. *Turner and Cirmena* suggested that NGS-based ctDNA assays demonstrated higher sensitivity and/or longer lead time compared to ddPCR assays within the same cohort (Table S1).

We mined raw data from the 12 studies and grouped cases into three molecular subtypes: HER2^+^, HR^+^HER2^−^, and TNBC (Fig. 1B). In the TNBC cohort, ctDNA detection yielded an average sensitivity of 81.2% (41/50 cases; 95% CI: 78.4%–84.0%), an average specificity of 63.1% (77/123 cases; 95% CI: 61.4%–64.8%), and an average lead time of 10.7 months (18 cases; 95% CI: 10.0–11.4) (Fig. 1B). In contrast, in the HER2^+^ cohort, the average sensitivity was 81.9% (12/13 cases; 95% CI: 78.4%–85.4%), the average specificity was 90.4% (85/93 cases; 95% CI: 86.6%–94.1%), and the average lead time was 17.7 months (12 cases; 95% CI: 16.0–19.5). Similarly, in the HR^+^HER2^−^ cohort, the average sensitivity was 75.8% (42/51 cases; 95% CI: 74.3%–77.4%), the average specificity was 93.5% (170/181 cases; 95% CI: 91.6%–95.4%), and the average lead time was 17.7 months (42 cases; 95% CI: 17.0–18.5).

This observation prompted comparisons of lead time, sensitivity, and specificity of ctDNA tests across the HER2^+^, HR^+^HER2^−^, and TNBC cohorts. Strikingly, the lead time was shorter in the TNBC cohort than in the HER2^+^ and HR^+^HER2^−^ cohorts (Fig. 1C). While there was no significant difference in sensitivity among the three cohorts (Fig. 1D), the specificity was lower in the TNBC cohort than in the HER2^+^ and HR^+^HER2^−^ cohorts (Fig. 1E). Notably, the difference in specificity was largely influenced by *Radovich's* study, which was a real-world data. Overall, ctDNA testing performed less effectively in the TNBC cohort than in the HER2^+^ and HR^+^HER2^−^ cohorts. Our findings strongly suggest the need to refine ctDNA testing strategies for TNBC patients, which may include a personalized follow-up schedule with more visits.

ctDNA has shown potential as a predictive biomarker for tumor recurrence, not only in breast cancer but also in other cancer types. It can be measured using experimental techniques available in standard research laboratories,^5^ making it accessible to both researchers and clinicians. Our analysis suggests that ctDNA is a promising biomarker for predicting breast cancer recurrence, with acceptable sensitivity and specificity and the added merit of earlier detection. Nonetheless, its performance varies across subtypes and assay platforms, underscoring the need for integration with imaging, laboratory assays, and pathology in follow-up protocols to enable timely clinical decision-making. Importantly, our findings underpin the value of a personalized approach in applying ctDNA testing, particularly for TNBC patients.

## CRediT authorship contribution statement

**Luke F. Moat:** Writing – original draft, Validation, Investigation, Formal analysis, Data curation. **John G. Mayer:** Writing – original draft, Software, Investigation, Formal analysis, Data curation. **David S. Puthoff:** Writing – original draft, Investigation. **Adam M. Bissonnette:** Writing – original draft, Investigation. **Abdul R. Shour:** Resources. **Scott J. Hebbring:** Writing – original draft, Supervision, Methodology, Funding acquisition, Formal analysis. **Adedayo A. Onitilo:** Writing – original draft, Supervision, Funding acquisition, Conceptualization. **Zhi Wen:** Writing – original draft, Supervision, Project administration, Methodology, Investigation, Funding acquisition, Formal analysis, Data curation, Conceptualization.

## Funding

This work was supported by NCI grant CA189956 to A.A.O., NIGMS grant GM114128 to S.J.H., the Tom and Sally Ebenreiter Precision Medicine Research Award and Marshfield Clinic Research Foundation startup fund to Z.W.

## Conflict of interests

The authors declare no conflicts of interest.
