# Supplementary material for: Circulating tumor DNA predicts tumor recurrence in early-stage breast cancer: A meta-analysis
Source: Genes Dis. 2025 Oct 30;13(4):101908. doi: 10.1016/j.gendis.2025.101908 (PMC13011021; doi:10.1016/j.gendis.2025.101908)
Supplement: Multimedia component 1 [file mmc1.pdf]

Circulating Tumor DNA Predicts Tumor Recurrence in Early-Stage Breast Cancer: A Meta-Analysis

Luke F. Moat [1#], John G. Mayer [1,2#], David S. Puthoff [3#], Adam M. Bissonnette [1,4], Abdul R. Shour [3], Scott J. Hebbring [1], Adedayo A. Onitilo [3*], Zhi Wen [1*]

[1] Center for Precision Medicine Research, Marshfield Clinic Research Institute, Marshfield Clinic Health System, Marshfield, WI 54449, USA
[2] Office of Research Computing and Analytics, Marshfield Clinic Research Institute, Marshfield, WI 54449, USA
[3] Cancer Care and Research Center, Marshfield Clinic Research Institute, Marshfield Clinic Health System, Marshfield, WI 54449, USA
[4] Integrated Research & Development Lab, Marshfield Clinic Research Institute, Marshfield Clinic Health System, Marshfield, WI 54449, USA
[#] These authors contributed equally

*** Corresponding authors:**

Adedayo A. Onitilo, MD, PhD, MSCR, FACP
Medical Director of Oncology Service Line
Senior Research Scientist
Address: Cancer Care and Research Center
Marshfield Clinic Research Institute
1000 N Oak Ave, Room ML4810
Marshfield, WI 54449
Telephone: (715) 393-1400
e-mail: onitilo.adedayo@marshfieldclinic.org

Zhi Wen, MD & PhD
Associate Research Scientist
Address: Center for Precision Medicine Research
Marshfield Clinic Research Institute
1000 N Oak Ave, Room ML4810
Marshfield, WI 54449
Telephone: (715) 221-6444
e-mail: wen.zhi@marshfieldclinic.org

Table of Contents

Supplemental Materials and Methods

PRISM 2020 Checklist

Supplemental References

Supplemental Figures and Legends

Supplemental Materials and Methods

Definition of early-stage breast cancer

US National Cancer Institute includes ductal carcinoma in situ and stage I-IIIA breast cancers in early-stage breast cancer while Canadian Cancer Society regards the tumor at stages I-IIA as early-stage breast cancer. Moreover, UK Cancer Research defines that early breast cancer has not spread beyond the breast or the lymph nodes in the armpit on the same side of the body. We honored this variance between countries where the studies were conducted.

Literature search and inclusion criteria

To be included, studies must focus on early-stage breast cancer, report ctDNA detection results alongside tumor recurrence data, include a cohort size of at least 10 cases, and be written in English. Reviews, meta-analyses, and case reports were excluded.

We conducted a comprehensive search in PubMed to identify studies meeting our eligibility criteria. Additionally, we manually searched conference abstracts from the *Journal of Clinical Oncology*, *Cancer Research*, and *Annals of Oncology* to find further eligible studies. The final search was completed on January 19, 2025, without restrictions on publication year. Specific search strings were detailed below. Studies were initially screened by publication type, title, and abstract, followed by a full-text review for inclusion in the analysis. All records were independently reviewed by at least two researchers without the use of automation tools.

Study selection and data extraction

We extracted the raw data from 21 selected studies, focusing on cohort size, breast cancer molecular subtypes, sample collection timepoints, ctDNA examination techniques, assay sensitivity, specificity, and lead time for predicting clinically confirmed tumor recurrence.

The studies from References-27, 29, 30, 33, 37, 39 and 40 had been conducted in highly overlapped patient cohorts, and data from Reference-30 was chosen for supplemental Table-S2 because it had high case number and presented a superior overall performance of ctDNA test. The study from Reference-36 was excluded because of its highly irregular blood sample collection schedule. Studies from References-44 and 47 were excluded because of limited access to the raw data. All analyzed studies were screened for bias risk using the Quality in Prognosis Studies tool [1] by two researchers. Risk of bias assessments can be found in Supplemental Figure-S2.

Statistics

There are inherent variabilities in ctDNA detection techniques, breast cancer molecular subtypes, and the timing and frequency of blood draws across the included studies. To account for these differences, we excluded data obtained before the initiation of follow-up, as such measurements do not accurately represent the postsurgical tumor state. When multiple ctDNA assessments were available during follow-up, only the earliest detection was considered to avoid repeated counts for individual patients. Because this meta-analysis did not directly compare or validate assay platforms, all reported ctDNA techniques were analyzed collectively. Given the clinical relevance of molecular subtypes, patients were stratified into three cohorts for subgroup analyses. Collectively, these methodological adjustments ensured that the meta-analysis provided a robust evaluation of the sensitivity, specificity, and lead time of ctDNA assays across 12 harmonized studies in predicting tumor

recurrence in early-stage breast cancer after surgery. These methodological adjustments should be recognized when interpreting the pooled estimates.

Sensitivity referred to the probability that the ctDNA result was positive when tumor recurrence was present. It was calculated as: *Sensitivity = True Positive / (True Positive + False Negative)*. In contrast, specificity was the probability that the ctDNA result was negative when tumor recurrence was not present. It was calculated as: *Specificity = True Negative / (False Positive + True Negative)*. The lead time represented the time from the first detection of ctDNA during the follow-up period to the clinical confirmation of tumor recurrence. The average sensitivity, specificity, and lead time with 95% Confidence Intervals were calculated for all ctDNA assays in R statistical programming language, weighted by sample size of each study or molecular subtype.

A two-tailed chi-square test with two degree of freedom was performed to compare the sensitivity and specificity of ctDNA tests among TNBC, $HER2^+$, and HR^+HER2^- subtypes. A meta-analysis using the Metacont function of Meta package in R statistical programming language was employed to compare the lead times between the three subtypes with unequal variances, showing the mean difference, 95% Confidence Intervals and two-tailed *p* values. The Metacont function reports Cohen's Q values and I^2 test's results which are measures of heterogeneity. The tests did not show a significant result for heterogeneity, although the results for heterogeneity need to be interpreted with caution, given the small number of studies.

Limitations: Several factors may influence the outcomes of ctDNA studies, including variability in assay techniques, tumor molecular subtypes, and timing of blood draws. The frequency of blood collection during follow-up varied across studies—every 2, 3, 6, or 12 months—which may contribute to heterogeneity in the meta-analysis. Efforts to stratify patients by molecular subtypes were limited by small sample sizes and restricted access to raw data, preventing a comprehensive assessment of ctDNA performance across breast cancer subtypes. Despite these challenges, we systematically compared the sensitivity, specificity, and lead time of ctDNA techniques across studies using appropriate statistical models. The limited number of recurrence cases in the $HER2^+$ cohort (Figure-B) reduces the robustness of estimates for this subgroup. Larger, standardized prospective studies are needed to validate these findings and strengthen the reliability of conclusions for $HER2^+$ patients.

Data Availability Statement

All data were from publications. The authors have no more data to share.

PRISM 2020 Checklist

Page MJ, McKenzie JE, Bossuyt PM, et al. The PRISMA 2020 statement: an updated guideline for reporting systematic reviews. BMJ 2021;372:n71. This work is licensed under CC BY 4.0. To view a copy of this license, visit *https://creativecommons.org/licenses/by/4.0/*

Section and Topic	Item #	Checklist item	Location where item is reported
**TITLE**			
Title	1	Identify the report as a meta-analysis.	Title
**ABSTRACT**			
Abstract	2	See the PRISMA 2020 for Abstracts checklist.	Abstract
**INTRODUCTION**			
Rationale	3	Describe the rationale for the review in the context of existing knowledge.	Introduction
Objectives	4	Provide an explicit statement of the objective(s) or question(s) the review addresses.	Introduction
**METHODS**			
Eligibility criteria	5	Specify the inclusion and exclusion criteria for the review and how studies were grouped for the syntheses.	2.1
Information sources	6	Specify all databases, registers, websites, organisations, reference lists and other sources searched or consulted to identify studies. Specify the date when each source was last searched or consulted.	2.1, Figure 2
Search strategy	7	Present the full search strategies for all databases, registers and websites, including any filters and limits used.	2.1, Supp. Methods
Selection process	8	Specify the methods used to decide whether a study met the inclusion criteria of the review, including how many reviewers screened each record and each report retrieved, whether they worked independently, and if applicable, details of automation tools used in the process.	2.1, Supp. Methods
Data collection process	9	Specify the methods used to collect data from reports, including how many reviewers collected data from each report, whether they worked independently, any processes for obtaining or confirming data from study investigators, and if applicable, details of automation tools used in the process.	2.1
Data items	10a	List and define all outcomes for which data were sought. Specify whether all results that were compatible with each outcome domain in each study were sought (e.g. for all measures, time points, analyses), and if not, the methods used to decide which results to collect.	2.2, Table 1
	10b	List and define all other variables for which data were sought (e.g. participant and intervention characteristics, funding sources). Describe any assumptions made about any missing or unclear information.	NA
Study risk of bias assessment	11	Specify the methods used to assess risk of bias in the included studies, including details of the tool(s) used, how many reviewers assessed each study and whether they worked independently, and if applicable, details of automation tools used in the process.	Supp. Methods, Figure S2
Effect measures	12	Specify for each outcome the effect measure(s) (e.g. risk ratio, mean difference) used in the synthesis or presentation of results.	2.3
Synthesis methods	13a	Describe the processes used to decide which studies were eligible for each synthesis (e.g. tabulating the study intervention characteristics and comparing against the planned groups for each synthesis (item #5)).	2.3, Supp. Methods
	13b	Describe any methods required to prepare the data for presentation or synthesis, such as handling of missing summary statistics, or data conversions.	NA
	13c	Describe any methods used to tabulate or visually display results of individual studies and syntheses.	NA
	13d	Describe any methods used to synthesize results and provide a rationale for the choice(s). If meta-analysis was performed, describe the model(s), method(s) to identify the presence and extent of statistical heterogeneity, and software package(s) used.	2.3, Supp. Methods
	13e	Describe any methods used to explore possible causes of heterogeneity among study results (e.g. subgroup analysis, meta-regression).	NA
	13f	Describe any sensitivity analyses conducted to assess robustness of the synthesized results.	NA
Reporting bias assessment	14	Describe any methods used to assess risk of bias due to missing results in a synthesis (arising from reporting biases).	NA
Certainty assessment	15	Describe any methods used to assess certainty (or confidence) in the body of evidence for an outcome.	2.3
**RESULTS**			
Study selection	16a	Describe the results of the search and selection process, from the number of records identified in the search to the number of studies included in the review, ideally using a flow diagram.	3.1, Figure 2
	16b	Cite studies that might appear to meet the inclusion criteria, but which were excluded, and explain why they were excluded.	Table 2, Supp. Methods
Study characteristics	17	Cite each included study and present its characteristics.	Table 1
Risk of bias in studies	18	Present assessments of risk of bias for each included study.	Figure S2
Results of individual studies	19	For all outcomes, present, for each study: (a) summary statistics for each group (where appropriate) and (b) an effect estimate and its precision (e.g. confidence/credible interval), ideally using structured tables or plots.	Table 1
Results of syntheses	20a	For each synthesis, briefly summarise the characteristics and risk of bias among contributing studies.	Figure S2
	20b	Present results of all statistical syntheses conducted. If meta-analysis was done, present for each the summary estimate and its precision (e.g. confidence/credible interval) and measures of statistical heterogeneity. If comparing groups, describe the direction of the effect.	Table 2, Figure 3
	20c	Present results of all investigations of possible causes of heterogeneity among study results.	3.2
	20d	Present results of all sensitivity analyses conducted to assess the robustness of the synthesized results.	Table 2, Figure 3
Reporting biases	21	Present assessments of risk of bias due to missing results (arising from reporting biases) for each synthesis assessed.	ND
Certainty of evidence	22	Present assessments of certainty (or confidence) in the body of evidence for each outcome assessed.	Table 2, Figure 3
**DISCUSSION**			
Discussion	23a	Provide a general interpretation of the results in the context of other evidence.	Discussion
	23b	Discuss any limitations of the evidence included in the review.	Limitations
	23c	Discuss any limitations of the review processes used.	Limitations
	23d	Discuss implications of the results for practice, policy, and future research.	Discussion, conclusion
**OTHER INFORMATION**			
Registration and protocol	24a	Provide registration information for the review, including register name and registration number, or state that the review was not registered.	ND
	24b	Indicate where the review protocol can be accessed, or state that a protocol was not prepared.	ND
	24c	Describe and explain any amendments to information provided at registration or in the protocol.	NA
Support	25	Describe sources of financial or non-financial support for the review, and the role of the funders or sponsors in the review.	Funding
Competing interests	26	Declare any competing interests of review authors.	Conflict of Interest
Availability of data, code and other materials	27	Report which of the following are publicly available and where they can be found: template data collection forms; data extracted from included studies; data used for all analyses; analytic code; any other materials used in the review.	Data Availability Statement

Supplemental References

1 Hayden JA, Cote P, Bombardier C. Evaluation of the quality of prognosis studies in systematic reviews. Ann Intern Med 2006; 144: 427-437.

2 Park W, Wei S, Kim BS, Kim B, Bae SJ, Chae YC et al. Diversity and complexity of cell death: a historical review. Exp Mol Med 2023; 55: 1573-1594.

3 Colleoni, M. et al. Annual Hazard Rates of Recurrence for Breast Cancer During 24 Years of Follow-Up: Results From the International Breast Cancer Study Group Trials I to V. J Clin Oncol 34, 927-935, doi:10.1200/JCO.2015.62.3504 (2016).

4 Fillon, M. Breast cancer recurrence risk can remain for 10 to 32 years. CA Cancer J Clin 72, 197-199, doi:10.3322/caac.21724 (2022).

5 Su, Y. H. et al. Human urine contains small, 150 to 250 nucleotide-sized, soluble DNA derived from the circulation and may be useful in the detection of colorectal cancer. J Mol Diagn 6, 101-107, doi:10.1016/S1525-1578(10)60497-7 (2004).

6 Saura, C. et al. Early-Stage Breast Cancer Detection in Breast Milk. Cancer Discov 13, 2180-2191, doi:10.1158/2159-8290.CD-22-1340 (2023).

7 Wan, J. C. M. et al. Liquid biopsies come of age: towards implementation of circulating tumour DNA. Nat Rev Cancer 17, 223-238, doi:10.1038/nrc.2017.7 (2017).

8 Thierry, A. R., El Messaoudi, S., Gahan, P. B., Anker, P. & Stroun, M. Origins, structures, and functions of circulating DNA in oncology. Cancer Metastasis Rev 35, 347-376, doi:10.1007/s10555-016-9629-x (2016).

9 Rykova, E. Y. et al. Cell-free and cell-bound circulating nucleic acid complexes: mechanisms of generation, concentration and content. Expert Opin Biol Ther 12 Suppl 1, S141-153, doi:10.1517/14712598.2012.673577 (2012).

10 Thakur, B. K. et al. Double-stranded DNA in exosomes: a novel biomarker in cancer detection. Cell Res 24, 766-769, doi:10.1038/cr.2014.44 (2014).

11 Thierry, A. R. et al. Origin and quantification of circulating DNA in mice with human colorectal cancer xenografts. Nucleic acids research 38, 6159-6175, doi:10.1093/nar/gkq421 (2010).

12 Diehl, F. et al. Circulating mutant DNA to assess tumor dynamics. Nature medicine 14, 985-990, doi:10.1038/nm.1789 (2008).

13 Ring, A., Nguyen-Strauli, B. D., Wicki, A. & Aceto, N. Biology, vulnerabilities and clinical applications of circulating tumour cells. Nat Rev Cancer 23, 95-111, doi:10.1038/s41568-022-00536-4 (2023).

14 Cohen, S. A., Liu, M. C. & Aleshin, A. Practical recommendations for using ctDNA in clinical decision making. Nature 619, 259-268, doi:10.1038/s41586-023-06225-y (2023).

15 Pantel, K. & Alix-Panabieres, C. Real-time liquid biopsy in cancer patients: fact or fiction? Cancer Res 73, 6384-6388, doi:10.1158/0008-5472.CAN-13-2030 (2013).

16 Ma, M. et al. "Liquid biopsy"-ctDNA detection with great potential and challenges. Ann Transl Med 3, 235, doi:10.3978/j.issn.2305-5839.2015.09.29 (2015).

17 Nader-Marta, G. et al. Circulating tumor DNA for predicting recurrence in patients with operable breast cancer: a systematic review and meta-analysis. ESMO Open 9, 102390, doi:10.1016/j.esmoop.2024.102390 (2024).

18 Arnedos, M. et al. Precision medicine for metastatic breast cancer--limitations and solutions. Nat Rev Clin Oncol 12, 693-704, doi:10.1038/nrclinonc.2015.123 (2015).

19 Oliveira, K. C. S. et al. Current Perspectives on Circulating Tumor DNA, Precision Medicine, and Personalized Clinical Management of Cancer. Mol Cancer Res 18, 517-528, doi:10.1158/1541-7786.MCR-19-0768 (2020).

20 Davidson, B. A., Croessmann, S. & Park, B. H. The breast is yet to come: current and future utility of circulating tumour DNA in breast cancer. Br J Cancer 125, 780-788, doi:10.1038/s41416-021-01422-w (2021).
21 Santonja, A. et al. Comparison of tumor-informed and tumor-naive sequencing assays for ctDNA detection in breast cancer. EMBO Mol Med 15, e16505, doi:10.15252/emmm.202216505 (2023).
22 Panet, F. et al. Use of ctDNA in early breast cancer: analytical validity and clinical potential. NPJ Breast Cancer 10, 50, doi:10.1038/s41523-024-00653-3 (2024).
23 Abbosh, C., Swanton, C. & Birkbak, N. J. Clonal haematopoiesis: a source of biological noise in cell-free DNA analyses. Ann Oncol 30, 358-359, doi:10.1093/annonc/mdy552 (2019).
24 Hu, Y. et al. False-Positive Plasma Genotyping Due to Clonal Hematopoiesis. Clinical cancer research : an official journal of the American Association for Cancer Research 24, 4437-4443, doi:10.1158/1078-0432.CCR-18-0143 (2018).
25 Marusyk, A. & Polyak, K. Tumor heterogeneity: causes and consequences. Biochimica et biophysica acta 1805, 105-117, doi:10.1016/j.bbcan.2009.11.002 (2010).
26 Pongor, L. S., Munkacsy, G., Vereczkey, I., Pete, I. & Gyorffy, B. Currently favored sampling practices for tumor sequencing can produce optimal results in the clinical setting. Sci Rep 10, 14403, doi:10.1038/s41598-020-71382-3 (2020).
27 Garcia-Murillas, I. et al. Mutation tracking in circulating tumor DNA predicts relapse in early breast cancer. Sci Transl Med 7, 302ra133, doi:10.1126/scitranslmed.aab0021 (2015).
28 Alba-Bernal, A. et al. Increased blood draws for ultrasensitive ctDNA and CTCs detection in early breast cancer patients. NPJ Breast Cancer 10, 36, doi:10.1038/s41523-024-00642-6 (2024).
29 Turner, N. C. et al. Results of the c-TRAK TN trial: a clinical trial utilising ctDNA mutation tracking to detect molecular residual disease and trigger intervention in patients with moderate- and high-risk early-stage triple-negative breast cancer. Ann Oncol 34, 200-211, doi:10.1016/j.annonc.2022.11.005 (2023).
30 Garcia-Murillas, I. et al. Assessment of Molecular Relapse Detection in Early-Stage Breast Cancer. JAMA Oncol 5, 1473-1478, doi:10.1001/jamaoncol.2019.1838 (2019).
31 Olsson, E. et al. Serial monitoring of circulating tumor DNA in patients with primary breast cancer for detection of occult metastatic disease. EMBO Mol Med 7, 1034-1047, doi:10.15252/emmm.201404913 (2015).
32 Elliott, M. J. et al. Ultrasensitive Detection and Monitoring of Circulating Tumor DNA using Structural Variants in Early-Stage Breast Cancer. Clinical cancer research : an official journal of the American Association for Cancer Research, doi:10.1158/1078-0432.CCR-24-3472 (2025).
33 Loi S; Johnston S; Arteaga C; Turner N, e. a. Abstract PS06-01: Results from a pilot study exploring ctDNA detection using a tumor-informed assay in the monarchE trial of adjuvant abemaciclib with endocrine therapy in HR+, HER2-, node-positive, high-risk early breast cancer. Cancer Research 84 (9_Supplement): PS06-01. (2024).
34 Shaw, J. A. et al. Serial Postoperative Circulating Tumor DNA Assessment Has Strong Prognostic Value During Long-Term Follow-Up in Patients With Breast Cancer. JCO Precis Oncol 8, e2300456, doi:10.1200/PO.23.00456 (2024).
35 Cutts, R. et al. Association of post-operative ctDNA detection with outcomes of patients with early breast cancers. ESMO Open 9, 103687, doi:10.1016/j.esmoop.2024.103687 (2024).

36 Cailleux, F. et al. Circulating Tumor DNA After Neoadjuvant Chemotherapy in Breast Cancer Is Associated With Disease Relapse. JCO Precis Oncol 6, e2200148, doi:10.1200/PO.22.00148 (2022).
37 Coakley, M. et al. Comparison of Circulating Tumor DNA Assays for Molecular Residual Disease Detection in Early-Stage Triple-Negative Breast Cancer. Clinical cancer research : an official journal of the American Association for Cancer Research 30, 895-903, doi:10.1158/1078-0432.CCR-23-2326 (2024).
38 Lipsyc-Sharf, M. et al. Circulating Tumor DNA and Late Recurrence in High-Risk Hormone Receptor-Positive, Human Epidermal Growth Factor Receptor 2-Negative Breast Cancer. J Clin Oncol 40, 2408-2419, doi:10.1200/JCO.22.00908 (2022).
39 Garcia-Murillas I, C. R., Turner N, et al. Ultra-sensitive ctDNA mutation tracking to identify molecular residual disease and predict relapse in patients with early breast cancer. Journal of Clinical Oncology Volume 42, Number 16_suppl (2024).
40 Garcia-Murillas, I. et al. Longitudinal monitoring of circulating tumor DNA to detect relapse early and predict outcome in early breast cancer. Breast Cancer Res Treat 209, 493-502, doi:10.1007/s10549-024-07508-2 (2025).
41 Nguyen Hoang, V. A. et al. Genetic landscape and personalized tracking of tumor mutations in Vietnamese women with breast cancer. Mol Oncol 17, 598-610, doi:10.1002/1878-0261.13356 (2023).
42 Barnell, E. K. et al. Personalized ctDNA micro-panels can monitor and predict clinical outcomes for patients with triple-negative breast cancer. Sci Rep 12, 17732, doi:10.1038/s41598-022-20928-8 (2022).
43 Janni W, H. J., Braun T, Huesmann S, et al. Abstract 3403: Multiomic, plasma-only circulating tumor DNA(ctDNA) assay identifies breast cancer patients with minimalresidual disease (MRD) and predicts distant recurrence. Cancer Research 82 (12_Supplement): 3403. (2022).
44 Elliott M, F. A., Dou A, CesconD, et al. Longitudinal evaluation of circulating tumour DNA in early breast cancer using a plasma-only methylation-based assay. Annals of Oncology Volume 34, Supplement 2S308 (2023).
45 Radovich, M. et al. Association of Circulating Tumor DNA and Circulating Tumor Cells After Neoadjuvant Chemotherapy With Disease Recurrence in Patients With Triple-Negative Breast Cancer: Preplanned Secondary Analysis of the BRE12-158 Randomized Clinical Trial. JAMA Oncol 6, 1410-1415, doi:10.1001/jamaoncol.2020.2295 (2020).
46 Cirmena, G. et al. Circulating Tumor DNA Using Tagged Targeted Deep Sequencing to Assess Minimal Residual Disease in Breast Cancer Patients Undergoing Neoadjuvant Chemotherapy. J Oncol 2020, 8132507, doi:10.1155/2020/8132507 (2020).
47 Zhang, X. et al. Parallel Analyses of Somatic Mutations in Plasma Circulating Tumor DNA (ctDNA) and Matched Tumor Tissues in Early-Stage Breast Cancer. Clinical cancer research : an official journal of the American Association for Cancer Research 25, 6546-6553, doi:10.1158/1078-0432.CCR-18-4055 (2019).
48 Dent, R. et al. Triple-negative breast cancer: clinical features and patterns of recurrence. Clinical cancer research : an official journal of the American Association for Cancer Research 13, 4429-4434, doi:10.1158/1078-0432.CCR-06-3045 (2007).
49 Magbanua, M. J. M. et al. Clinical significance and biology of circulating tumor DNA in high-risk early-stage HER2-negative breast cancer receiving neoadjuvant chemotherapy. Cancer Cell 41, 1091-1102 e1094, doi:10.1016/j.ccell.2023.04.008 (2023).
50 Dawson, S. J. et al. Analysis of circulating tumor DNA to monitor metastatic breast cancer. The New England journal of medicine 368, 1199-1209, doi:10.1056/NEJMoa1213261 (2013).

51 Chi, Y. et al. Dynamic analysis of circulating tumor DNA to predict the prognosis and monitor the treatment response of patients with metastatic triple-negative breast cancer: A prospective study. Elife 12, doi:10.7554/eLife.90198 (2023).
52 Tie, J. et al. Circulating tumor DNA as an early marker of therapeutic response in patients with metastatic colorectal cancer. Ann Oncol 26, 1715-1722, doi:10.1093/annonc/mdv177 (2015).
53 Medford, A. J. et al. Molecular Residual Disease in Breast Cancer: Detection and Therapeutic Interception. Clinical cancer research: an official journal of the American Association for Cancer Research 29, 4540-4548, doi:10.1158/1078-0432.CCR-23-0757 (2023).
54 Wen, Z. et al. Orphan nuclear receptor PNR/NR2E3 stimulates p53 functions by enhancing p53 acetylation. Mol Cell Biol 32, 26-35, doi:10.1128/MCB.05513-11 (2012).
55 Wen, Z. et al. Expression of Nras^{Q61R} and MYC transgene in germinal center B cells induces a highly malignant multiple myeloma in mice. Blood 137, 61-74, doi:10.1182/blood.2020007156 (2021).
56 Schwarzenbach, H., Hoon, D. S. & Pantel, K. Cell-free nucleic acids as biomarkers in cancer patients. Nat Rev Cancer 11, 426-437, doi:10.1038/nrc3066 (2011).
57 Ryan J. Sullivan, V. J. O. N., Kay Brinkmann, Daniel Enderle, Tina Koestler, Alexandra Spiel, Jennifer Emenegger, Mikkel Noerholm, Johan Skog, Carola Berking, and Keith Flaherty. Plasma-based monitoring of BRAF mutations during therapy for malignant melanoma using combined exosomal RNA and cell-free DNA analysis. Journal of Clinical Oncology Volume 33, Number 15_suppl (2015).

Figure S1

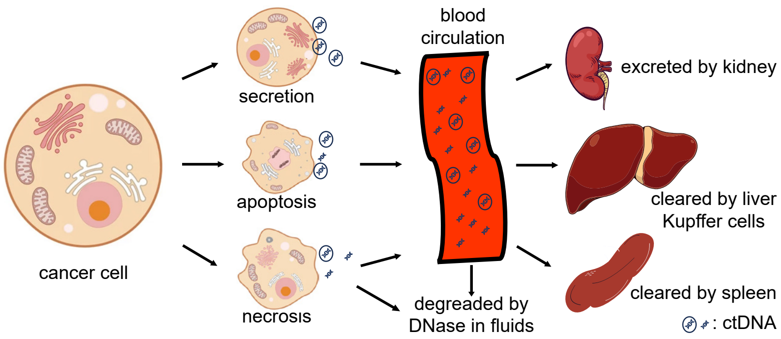

Figure S1: Illustration of ctDNA biology. The pictures of cells were obtained from the paper [2], and the pictures of organs were free from www.freepik.com. DNA exists inside nucleus and mitochondria as well as freely outside of cells. The extracellular DNA is cell-free DNA (cfDNA) that can enter both the circulating fluids (e.g., blood) and other fluids (e.g., urine and breast milk) [5-7]. Circulating tumor DNA (ctDNA) originates from tumor cells and is a type of cfDNA. Usually, tumor cells present very dynamic biological activities, such as apoptosis, necrosis, secretion, etc. [8]. Consequently, ctDNA has been detected in apoptotic bodies, exosomes and microvesicles in the circulating blood. The ctDNA usually is cleared by liver, kidney and spleen.

Figure S2

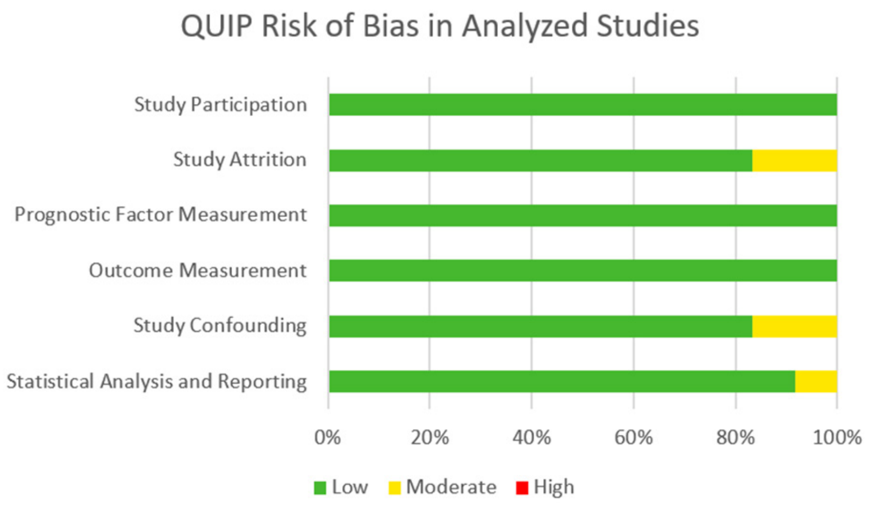

Figure S2: Risk of bias assessment results using the Quality in Prognosis Studies (QUIP) tool for all analyzed studies.

Figure S3

A

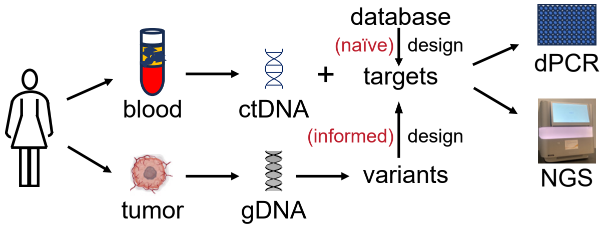

B

Class	Assay name	Time	Technology	Targets	Prerequisites	CHIP
Informed	ddPCR	4 weeks	droplet digital PCR	genomics (≥1 somatic variants)	Sequencing paired tumor-normal tissues for targets design	Excluded
	Pathlight	4 weeks	Multiplex digital PCR	Genomics (16 somatic structural variants)		
	Signatera [a]	5 weeks	PCR amplicon-based NGS	genomics (top 16 somatic variants)		
	RaDaR [a]	5 weeks		genomics (≤ 48 somatic variants)		
	Invitae	5 weeks		genomics (18 - 50 variants)		
	NeXT Personal Dx [b]	5 weeks	Hybridization capture-based NGS	genomics, transcriptomics (≤1800 somatic & germline variants)		
Naïve	ddPCR	5 days	droplet digital PCR	genomics (≥ 1 somatic variants)	Pre-designed targets of interest	No
	Guardant Reveal	10 days	Hybridization capture-based NGS	methylation, genomics	Pre-designed pan-cancer panel	Excluded
	Foundation One Liquid CDx [c]	10 days		genomics (311 variants)		No
	tTDS	10 days	PCR amplicon-based NGS	genomics (>152 somatic variants and copy number variants)	Commercial panels	
	Home-made NGS	10 days		genomics (somatic variants of 204 genes)	Gene panels are designed based on COSMIC database	

[a] Breakthrough Device Designations granted by FDA; [b] Medicare Coverage for NeXT Dx(R); [c] FDA-approved companion diagnostic indications include breast cancer.
gDNA: genomic DNA; dPCR: digital polymerase chain reaction; ddPCR: droplet dPCR; NGS: next-generation deep sequencing;
CHIP: clonal hematopoiesis of indeterminate potential; tTDS: Tagged targeted deep sequencing.

Figure S3: Summary of ctDNA assays used to detect residual disease or tumor recurrence of early-stage breast cancer. (A) Illustration of ctDNA assays. (B) Available ctDNA assays. The assays can be either informed or naïve [20, 21]. “Informed” refers to designing the targets of detection upon the somatic variants in each primary breast cancer sample. To do this, the residual tumor tissues and their paired normal tissues will be processed for next-generation deep sequencing (NGS), such as whole exome sequencing, through which the somatic variants will be identified and prioritized to be the targets of detection. In contrast, “Naïve” refers to pre-designing the targets of detection upon the variants commonly identified throughout the patient population of one tumor type or multiple tumor types [22]. The merits of informed assays include the personalized tracking of the limited amount of ctDNA and the recognition of contaminations of variants in cfDNA originating from clonal hematopoiesis of indeterminate potential (CHIP) and other normal tissues [23, 24]. However, the informed assays are determined by residual tumor tissues and sampling methods, which may lead to an incomplete presentation of tumor heterogeneity [25, 26]. On the contrary, conducting buffy coat analysis of leukocyte gDNA from the same blood sample can alleviate the contamination of cfDNA in the naïve assays. Generally, the turnaround time of the naïve assays is much shorter than that of the informed assays, mainly due to the procedures to identify the targets of detection in the informed assays.

Table S1

Study	Sample size of early-stage breast cancer	Time points of blood draw				ctDNA Methods	Sensitivity	Specificity	Ave. time (ctDNA^{+} to recurrence)	Class
		T0	T1	T2	T3 / follow-up					
Shaw, *et al*	140 (23 TNBC, 42 HER2^{+}, 75 HR^{+}HER2^{-}) [34]	Yes	No	No	every 6 mo for 12 years	Signatera (up to 16 patient-specific, somatic, clonal SNVs)	24/30 (80%)	105/110 (95.5%)	20.3 mo	informed
Loi, *et al*	178 (178 HR^{+}HER2^{-} node^{+}high-risk) [33]	No	No	No	Day-1 and Day-731 of treatment		42/70 (60.0%)	108/108 (100%)	n/a	
Cailleux, *et al*	43 (17 HER2^{-}, 11 HER2^{+}, 12 TNBC) [36]	Yes	No	Pre	Yes (various frequencies)		4/6 (66.7%) [a]	33/34 (97.1%) [a]	3.3 mo [a]	
Cutts, *et al*	17 (1 TNBC, 3 HER2^{+}, 13 HR^{+}HER2^{-}) [35]	No	Yes	Post	Yes		0/1 (0%)	13/16 (81.3%)	n/a	
Garcia-Murillas, *et al*	76 (23 TNBC, 33 HER2^{+}, 16 HR^{+}HER2^{-}, 4 n/a) [39]	Yes	cycle-2	Post	every 3 mo for Year 1, every 6 mo for Year 2-10	NeXT Personal Dx	10/10 (100%)	58/61 (95.1%)	12.5 mo	
	61 (16 TNBC, 20 HER2^{+}, 21 HR^{+}HER2^{-}, 4 n/a) [40]	Yes	No	No	every 3 mo for Year 1-2, every 6 mo for Year 3-5	Invitae (11-50 variants)	11/13 (84.6%)	43/48 (89.6%)	11.7 mo	
	55 (14 TNBC, 21 HER2^{+}, 20 HR^{+}HER2^{-}, 43 follow-up) [27]	Yes	No	Post	every 6 mo until relapse	ddPCR (1-4 SNVs)	12/15 (80.0%)	27/28 (96.4%)	7.9 mo	
	101 (25 TNBC, 41 HER2^{+}, 35 HR^{+}HER2^{-}) [30]	No	No	No	every 3 mo for Year 1, every 6 mo for Year 2-5	ddPCR	9/12 (75%)	82/89 (92.1%)	10.7 mo	
Olsson, *et al*	20 (1 TNBC, 17 HR^{+}HER2^{-}, 2 HR^{+}HER2^{+}) [31]	Yes	No	No	3rd, 8^{th,} 12th, 24th and 36th month	ddPCR (6-19 SNVs)	13/14 (92.9%)	6/6 (100%)	11 mo	
Elliott, *et al*	77 (35 HER2^{+}, 18HR^{+}HER2^{-}, 24 TNBC) [32]	Yes	Yes	Peri	Yes	Multiplex dPCR (16 structural variants)	17/19 (89.5%)	57/58 (98.3%)	13.9 mo	
Alba-Bernal, *et al*	15 (6 TNBC, 7 HER2^{+}, 2 HER2^{-}) [28]	Yes	Post	1 mo post	every 6 mo until relapse	ddPCR (1 SNV)	2/2 (100%)	7/13 (53.8%)	9 mo	
Hoang, *et al*	32 (16 HER2^{-}, 15 HER2^{+}, 1 TNBC) [41]	No	No	Pre	every 6 mo	Homemade NGS (2-27 variants)	3/3 (100%)	25/29 (86.2%)	10 mo	
Barnell, *et al*	50 (50 TNBC) [42]	Yes	Yes	On	every 3-6 mo for 5 years	Homemade NGS (4-6 variants)	4/5 (80%)	n/a	9.9 mo	
Turner, *et al*	161 (161 TNBC, 14 in observation group) [29]	Yes	Pre	Post	every 3 mo for Year -1, every 6 mo for Year 2-6	ddPCR (1-2 SNVs)	n/a	n/a	4.1 mo	
	114 (114 TNBC) [37]					RaDaR (33-56 variants ddPCR (1-2 SNVs)	45/47 (95.7%)	61/67 (91.0%)	6.1 mo	
Lipsyc, *et al*	83 (83 HR^{+}HER2^{-}) [38]	No	No	No	every 6-12 mo	RaDaR (12-51 variants)	6/7 (85.7%)	74/76 (97.4%)	16.0 mo	
Cirmena, *et al*	10 (2 HR^{+}HER2^{+}, 8 HR^{+}HER2^{-}) [46]	Yes	No	No	every 24 weeks for 2 years	ddPCR (1 SNV)	1/4 (25%)	4/6 (66.7%)	6 mo	naïve
						tTDS (>152 events)	3/4 (75%)	4/6 (66.7%)	6 mo	
Radovich, *et al*	112 (112 TNBC) [45]	No	No	No	peri-adjuvant treatment	Foundation One Liquid Assay (68-77 variants)	23/29 (79.3%)	40/83 (48.2%)	n/a	
Janni, *et al*	38 [43]	No	No	No	12-36 mo post-diagnosis or at clinical recurrence	Guardant Reveal	12/20 (60.0%)	15/18 (83.3%)	n/a	
Elliott, *et al*	83 (38 HR^{+}HER2^{-}, 45 TNBC) [44]	Yes	Yes	Post	Yes	Guardant Reveal	n/a	62/66 (93.9%)	n/a	
Zhang, *et al*	102 (18 TNBC, 27 HER2^{+}, 50 HR^{+}HER2^{-}, 7 n/a) [47]	No	No	Pre	every 4-6 mo	Homemade NGS (SNVs of 204 genes)	n/a	n/a	n/a	

T0: baseline before treatment; T1: non-adjuvant treatment; T2: surgery (plus radiotherapy in some cases); T3/follow-up: post-surgery adjuvant treatments and follow-up.
Ave. time (ctDNA^{+} to recurrence): average lead time (months) from the first detection of ctDNA to clinical confirmation of relapse.
[a] The follow-up schedules were not fixed, leading to a less-reliable sensitivity, specificity and lead time.
Red font: data during the follow-up periods were analyzed; *green font*: data at all the timepoints were analyzed.

Table S1: Summary of studies using ctDNA assays to detect residual disease or tumor recurrence of early-stage breast cancer. The 21 publications consisted of 16 informed and 5 naïve studies to detect ctDNA in plasma collected during the follow-up period. 6 studies chose ddPCR to detect ctDNA at multiple timepoints, including baseline at diagnosis (T0), non-adjuvant treatment (T1), surgery (T2) or adjuvant treatment (T3)/follow-up, among the informed group [27-32]. The researchers tracked 1-2 variants at most of the cases and up to 19 variants in some cases. The informed group also included 4 Signatera [33-36], 2 RaDaR [37, 38], 1 NeXT Personal Dx [39], 1 Invitae studies [40] and 2 home-made NGS [41, 42], all of which simultaneously tracked many variants per NGS test (Table-1). On the other hand, 2 Guardant Reveal [43, 44], 1 Foundation One Liquid [45], 1 ddPCR [46], 1 home-made NGS [47], and 1 tTDS [46] were conducted in the naïve group.

Table S2

Study	Sample size of early-stage breast cancer	Blood draw (T3 / follow-up)	Sensitivity	Specificity	Average lead time ctDNA$^+$ to recurrence
Shaw, *et al*	140 (23 TNBC, 42 HER2$^+$, 75 HR$^+$HER2$^-$)	every 6 mo for 12 years	24/30 (80%)	105/110 (95.5%)	20.3 mo (20 cases)
Cutts, *et al*	17 (1 TNBC, 3 HER2$^+$, 13 HR$^+$HER2$^-$)	Yes	0/1 (0%)	13/16 (81.3%)	n/a
Garcia-Murillas, *et al*	101 (25 TNBC, 41 HER2$^+$, 35 HR$^+$HER2$^-$)	every 3 mo for Year 1, every 6 mo for Year 2-5	9/12 (75%)	82/89 (92.1%)	10.7 mo (9 cases)
Olsson, *et al*	20 (1 TNBC, 17 HR$^+$HER2$^-$, 2 HR$^+$HER2$^+$)	3rd, 8^{th,} 12th, 24th and 36th month	13/14 (92.9%)	6/6 (100%)	11 mo (13 cases)
Elliott, *et al*	77 (35 HER2$^+$, 18 HR$^+$HER2$^-$, 24 TNBC)	Yes	17/19 (89.5%)	57/58 (98.3%)	13.9 mo (17 cases)
Alba-Bernal, *et al*	15 (6 TNBC, 7 HER2$^+$, 2 HER2$^-$)	every 6 mo until relapse	2/2 (100%)	7/13 (53.8%)	9 mo (2 cases)
Hoang, *et al*	32 (16 HER2$^-$, 15 HER2$^+$, 1 TNBC)	every 6 mo	3/3 (100%)	25/29 (86.2%)	10 mo (3 cases)
Barnell, *et al*	50 (50 TNBC)	every 3-6 mo for 5 years	4/5 (80%)	n/a	9.9 mo (4 cases)
Lipsyc, *et al*	83 (83 HR$^+$HER2$^-$)	every 6-12 mo	6/7 (85.7%)	74/76 (97.4%)	16.0 mo (6 cases)
Cirmena, *et al*	10 (2 HR$^+$HER2$^+$, 8 HR$^+$HER2$^-$)	every 6 mo for 2 years	3/4 (75%)	4/6 (66.7%)	6 mo (3 cases)
Radovich, *et al*	112 (112 TNBC)	peri-adjuvant treatment	23/29 (79.3%)	40/83 (48.2%)	n/a
Janni, *et al*	38	12-36 mo post-diagnosis, at clinical recurrence	12/20 (60.0%)	15/18 (83.3%)	n/a
Overall			116/146 (80.7%) [95% CI: 80.3-81.1]	428/504 (78.1%) [95% CI: 77.6-78.5]	15.5 mo (77 cases) [95% CI: 15.1-15.9]

Average was calulated after weighted by the sample size or case number in each study. *CI:* Confidence Interval. The studies from References-27, 29, 30, 33, 37, 39 and 40 had been conducted in highly overlapped patient cohorts, and data from Reference-30 was chosen because of its high case number and overall performance. The study from Reference-36 was excluded because of its highly irregular blood sample collection schedule. Studies from References-44 and 47 were excluded because of limited access to the raw data.

Table S2: Overall sensitivity, specificity and lead time of ctDNA assays to predict tumor recurrence of early-stage breast cancer.
